# Estrogen receptor (ER) was regulated by RNPC1 stabilizing mRNA in ER positive breast cancer

**DOI:** 10.18632/oncotarget.3654

**Published:** 2015-03-26

**Authors:** Liang Shi, Tian-Song Xia, Xiao-Long Wei, Wenbin Zhou, Jinqiu Xue, Lin Cheng, Peipei Lou, Chunlian Li, Ying Wang, Ji-Fu Wei, Qiang Ding

**Affiliations:** ^1^ Jiangsu Breast Disease Center, The First Affiliated Hospital with Nanjing Medical University, Nanjing, China; ^2^ Research Division of Clinical Pharmacology, The First Affiliated Hospital with Nanjing Medical University, Nanjing, China; ^3^ Department of Pathology, Cancer Hospital of Shantou University Medical College, Shantou, China

**Keywords:** estrogen receptor, RNPC1, mRNA stability, breast cancer

## Abstract

Estrogen receptors (ERs), including ERα and ERβ, mainly mediate the genotype effect of estrogen. ERα is highly expressed in most breast cancers. Endocrine therapy is the most effective and safety adjunctive therapy for ER positive breast cancers. RNPC1, an RNA binding protein (RBP), post-transcriptionally regulating gene expression, is emerging as a critical mechanism for gene regulation in mammalian cells. In this study, we revealed RNPC1's capability of regulating ERα expression. There was a significant correlation between RNPC1 and ERα expression in breast cancer tissues. Ectopic expression of RNPC1 could increase ERα transcript and expression in breast cancer cells, and vice versa. Consistent with this, RNPC1 was able to bind to ERα transcript to increase its stability. Furthermore, overexpression of ERα could decrease the level of RNPC1 transcript and protein. It suggested a novel mechanism by which ERα expression was regulated via stabilizing mRNA. A regulatory feedback loop between RNPC1 and ERα was proved. It indicated that RNPC1 played a crucial role in ERα regulation in ER-positive breast cancers via binding to ERα mRNA. These findings might provide new insights into breast cancer endocrine therapy and ERα research.

## INTRODUCTION

The incidence of female breast cancer increases rapidly in recent years and poses an enormous threaten to women's health [1]. It still contributes to the most cancer death cases in women even though great advances in diagnosis and therapy in breast cancer have been achieved [2].

Estrogen is the essential hormone for mammary gland growth and development, but high level of estrogen is a major risk factor for breast cancer [3-5]. Two possible mechanisms have been proposed to explain the increased risk: (1) estrogen receptor (ER) mediated stimulation of breast cell proliferation with a concomitant enhanced rate in DNA mutations [6] and (2) metabolism of estradiol to genotoxic metabolites, such as estradiol-adenine-guanine adducts and oxygen free radicals, resulting in the increase of DNA mutations [5].

As receptors of estrogen, ERs mainly mediate the genotype effect of estrogen [7-8]. They act as nuclear transcriptional regulators of multiple target genes [9]. The two structurally related ERs, ERα and ERβ, are the products of two separate genes and show distinct distributions and functions [7]. Only ERα is essential for breast development and activates pro-proliferative signaling in normal breast and breast cancers, whereas ERβ generally antagonizes ERα in the breast [10, 11]. The classic effects of ERs regulating gene expression are recruiting cofactors and binding to an estrogen-responsive element (ERE) in the nucleus [12, 13]. Compared with those in normal breast tissues, the expression of ERα is increased, while ERβ is reduced in breast cancer [14-16]. 70% of breast cancers express ERα and are classified as estrogen receptor positive (ER-positive). In clinical practice, ERα is a well-established diagnostic and prognostic marker in breast cancer. For example, the breast cancer patients with ER negative have shorter survival [17]. The patients, who are diagnosed with ER-positive, are suitable candidates for hormonal therapies, which aim to block estrogen stimulation of breast cancer cells. The selective ER modulators (SERMs) compete for the binding of estrogen to the receptor and result in the inhibition of hormone action. Tamoxifen, a SERM, is the standard endocrine treatment for ER-positive breast cancers. On the other hand, the aromatase inhibitors (AIs), which block the synthesis of estrogen, are also widely used. The patients with ERα positive tumors can widely benefit from these endocrine therapies [18-21].

Given the key role of ERα in breast cancer, the knowledge of mechanisms in expression and regulation of ERα makes great sense in the battle against this disease. Upon binding of estrogen, ERα can react to DNA regulatory elements and activate or repress its target genes expression [22]. To prevent inappropriate transcription events, ERα activity is tightly regulated by several mechanisms. Firstly, N-terminal estrogen-independent and C-terminal estrogen-dependent transactivation function domains (AF1 and AF2, respectively) contribute to the transcription of ERα [23, 24]. Secondly, estrogen stimulates endogenous ER express through membrane-initiated signaling pathways [25]. For example, the kinase cascades, calcium and other second messengers impact the transcription in the nucleus [26]; the activation of ERK or PI3 kinase promotes G1/S cell cycle progression [27]. Thirdly, the post-translational modifications of ERα have been demonstrated for phosphorylation, acetylation and sumoylation [28]. Fourthly, the stability of ERα protein can be regulated through the ubiquitin-proteasome pathway [29, 30]. However, from transcription to protein level of ERα, whether or how ERα is regulated by mRNA level has not been revealed. In our previous study, RNPC1, instead of RNPC1a, also called RBM38, was found expressed in breast cancer and had a potential function on playing a tumor-suppressor role [31].

Given the strong evidence that RNPC1 and ERα expression are positively related in clinic breast cancer specimen, it suggested RNPC1 could be a novel mechanism regulating ERα. We are trying to prove the relevance of these two molecules and reveal the details of their interaction.

## RESULTS

### Immunohistochemical (IHC) staining of RNPC1 in human breast cancer tissues

**Figure 1:**
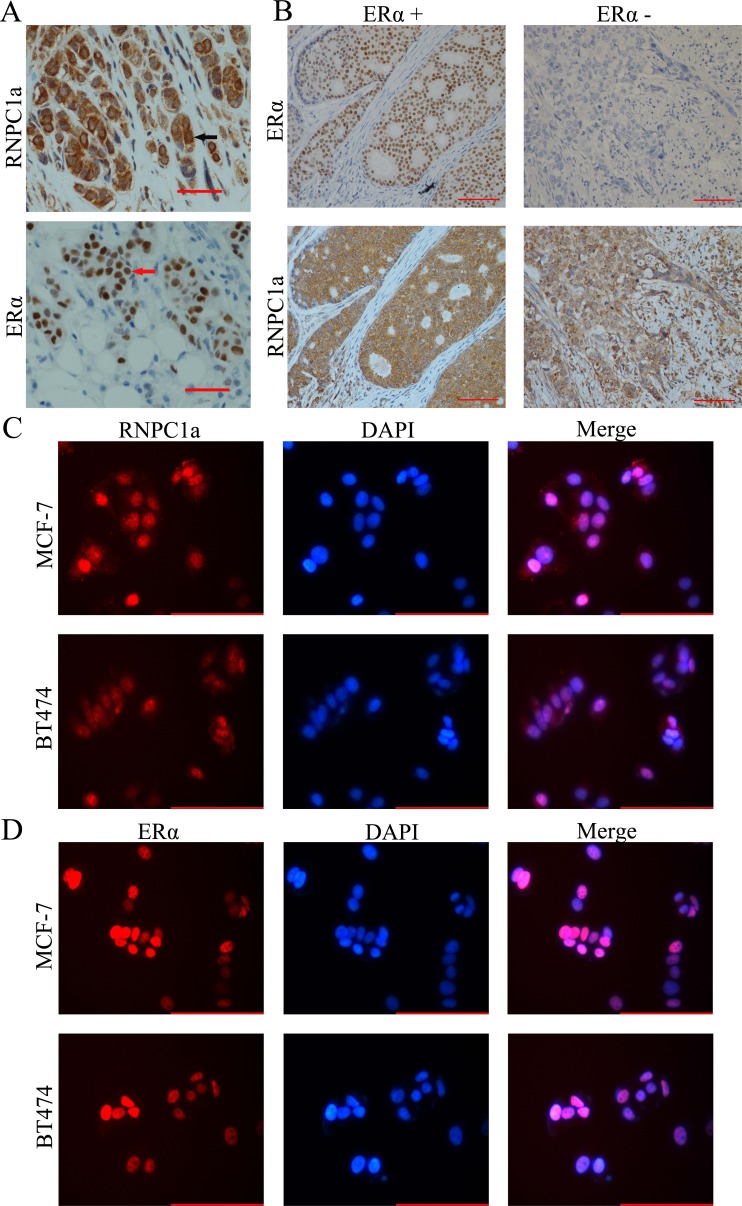
RNPC1 expression correlated with ERα positive human breast cancer (**A**) IHC analysis of RNPC1a and ERα in breast cancer at 200× magnification. RNPC1a was mainly expressed in the cytoplasm (arrowed) and ERα was mainly expressed in the nucleus (arrowed). Scale bars indicate 100 μm. (**B**) ERα positive breast cancer expressed high level of RNPC1a; ERα negative breast cancer expressed low level of RNPC1. Scale bars indicate 100 μm. (**C**) Immunofluorescence staining of RNPC1a in MCF-7 and BT474 cells at 400× magnification. Red represented RNPC1a staining. Blue signals represented nuclear DNA staining with DAPI. Scale bars indicate 100 μm. (**D**) Immunofluorescence staining of ERα in MCF-7 and BT474 cells. Red represented ERα staining. Blue signals represented nuclear DNA staining with DAPI. Scale bars indicate 100 μm.

To confirm the expressive level of RNPC1a in breast cancer, IHC analysis was performed to investigate the expression of RNPC1a in 90 breast cancer tissues. RNPC1a was mainly expressed in the cytoplasm and ERα was mainly expressed in the nucleus (Figure 1A). The correlation between RNPC1a expression and clinicopathological features was analyzed (Table 1). RNPC1a expression was obviously higher in ERα positive breast cancers compared with ERα negative breast cancers (p < 0.01). The representative images of RNPC1a expression in ERα positive and negative breast cancer tissues were showed in Figure 1B. It indicated that RNPC1a expression was significantly correlated with ERα in breast cancer. To clarify the celluer location of RNPC1a and ERα, immunofluorescence was applied to detect the distribution of RNPC1a and ERα in breast cancer cells. RNPC1a was mainly expressed in the nucleus and cytoplasm in MCF-7 (Figure 1C) cells, and ERα was mainly expressed in the nucleus (Figure 1D). These phenomena were also observed in BT474 cells (Figure 1C and 1D).

**Table 1:** Association of RNPC1 with ERα and clinicopathological characteristics of breast cancer

Clinicopathologicalcharacteristics	RNPC1a expression			
	No. of cases	Low (%)	High (%)	P-value
Age				0.143
<50	48	28(58.33)	20(41.67)	
≥50	42	18(42.86)	24(57.14)	
pathological grade				0.396
I-II	67	36(53.73)	31(46.27)	
III	23	10(43.48)	13(56.52)	
TNM stage				0.625
I-II	81	42(51.85)	39(48.15)	
III	9	4(44.44)	5(55.56)	
ERα				0.001
negative	38	27(71.05)	11(28.95)	
positive	52	19(36.54)	33(63.46)	

ERα: estrogen receptor α.

TNM stage is according to the Union Internationale Contre le Cancer criteria.

### ERα expression was increased by ectopic expression of RNPC1

**Figure 2:**
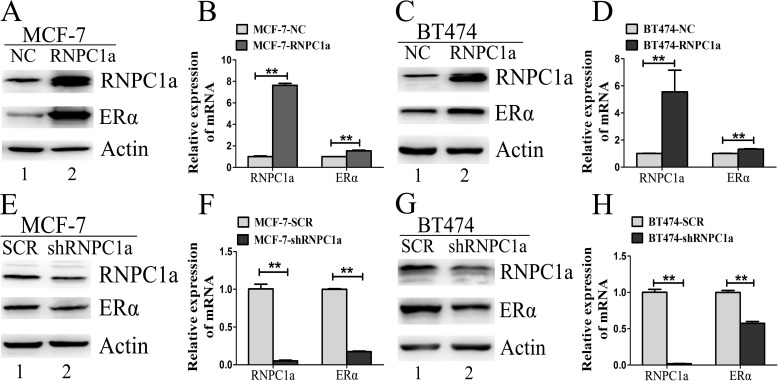
ERα expression was influenced by RNPC1 in ER positive breast cancer cells (A-D) The level of ERα protein was increased by overexpression of RNPC1a in MCF-7 (A, B) and BT474 (C, D). (A, B) MCF-7 cells were transfected with lentivirus containing either control luciferase (NC) or RNPC1a overexpression (RNPC1a). (**A**) Western blot and (**B**) qRT-PCR were used to analyze the expression of RNPC1a and ERα. (C, D) The experiment shown in panel A was also performed in BT474 cells. (**C**) Western blot and (**D**) qRT-PCR were used to analyze the expression of RNPC1a and ERα. (E-H) The level of ERα protein was reduced by knockdown of RNPC1a in MCF-7 (E, F) and BT474 (G, H) cells. (E, F) MCF-7 cells were transfected with a control (SCR) and RNPC1a knockdown (shRNPC1a) lentivirus. (**E**) Western blot and (**F**) qRT-PCR were used to analyze the expression of RNPC1a and ERα. (G, H) The experiment shown in panel E was also performed in BT474 cells. (**G**) Western blot and (H) qRT-PCR were used to analyze the expression of RNPC1a and ERα. The relative quantification was calculated by the ΔΔCt method and normalized based on β-actin. Data were means of three separate experiments and presented as mean ± SEM, **p < 0.01.

MCF-7 cells were transfecfed with lentivirus to overexpress RNPC1a and the control. ERα expression was obviously increased in MCF-7 cells after RNPC1a up-regulated both in protein and RNA levels (Figure 2A and 2B, p < 0.01). The same result was also observed in BT474 cells with lower rising level (Figure 2C and 2D, p < 0.01). In ER negative breast cancer cells MDA-MB-231 and SUM 1315, there was no ERα expression after RNPC1a over-expression (Figure S1A-D, p < 0.01), suggesting that RNPC1a could not affect ERα state in ER negative breast cancers.

### RNPC1 down-regulation decreased ERα expression in ER positive breast cancer cells

To verify endogenous RNPC1a can regulate ERα expression, RNPC1a was knockdown in MCF-7, BT474, MDA-MB-231 and SUM 1315. ERα protein and transcript levels in MCF-7 (Figure 2E and 2F, p < 0.01) and BT474 (Figure 2G and 2H, p < 0.01) were significantly decreased. However, the protein levels of ERα couldn't be detected in MDA-MB-231 and SUM 1315 (Figure S1E and S1G). The transcripts of RNPC1a and ERα in MDA-MB-231 (Figure S1F, < 0.01) and SUM 1315 (Figure S1H, < 0.01) consisted with protein expression. It indicated that RNPC1a could positively affect ERα expression in ER positive breast cancers.

### RNPC1 showed no influence on ERβ expression in breast cancer cells

It is also very important to figure out whether RNPC1a have influence on ERβ. There was no change of ERβ expression in protein (Figure S2A and S2C) and transcript levels (Figure S2B and S2D) after RNPC1a overexpressed in MCF-7 and BT474. In addition, when RNPC1a was knockdown, there was also no significant alteration of ERβ expression in protein level (Figure S2E and S2G) or transcript levels (Figure S2F and S2H) in MCF-7 and BT474 cells. It indicated that RNPC1a could not influence ERβ expression in breast cancer cells.

### RNPC1 could bind to ERα transcript and increase its stability

**Figure 3:**
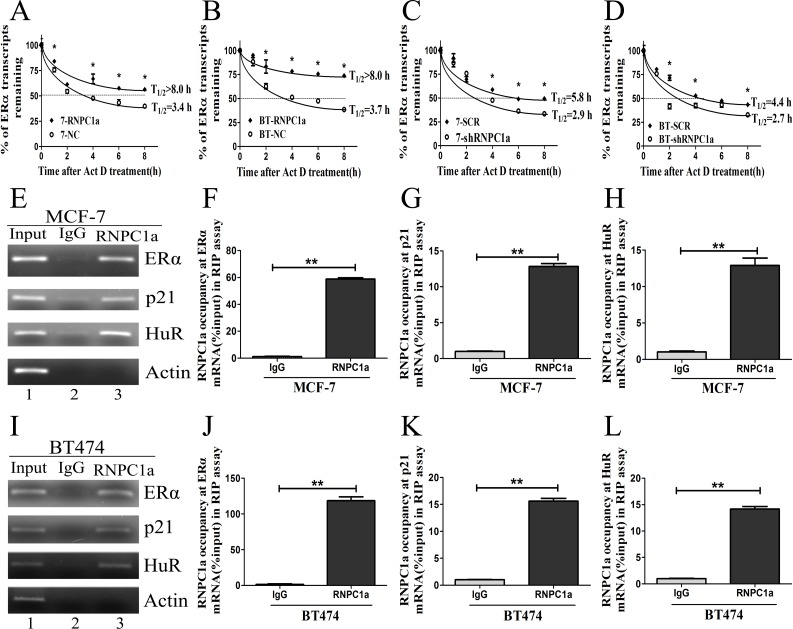
RNPC1 could bind to ERα transcript and enhance its stability (**A**, **B**) The half-life of ERα transcript was enhanced by RNPC1a overexpression. (A) MCF-7 (7) and (B) BT474 (BT) were transfected with lentivirus to overexpress RNPC1a. The control (NC) and RNPC1a overexpression (RNPC1a) cells were treated with 5ug/ml actinomyclin D (Akt D) for 0, 1, 2, 4, 6, 8h. The relative quantification was calculated by the ΔΔCt method and normalized based on β-actin. (**C**, **D**) The half-life of ERα transcript was decresed after RNPC1a knockdown. (C) MCF-7 (7) and (D) BT474 (BT) were transfected with the negative control vectors (SCR) and RNPC1a knockdown lentivirus (shRNPC1a). The following experiments were conducted according to those in RNPC1a overexpression. (**E**-**L**) RNPC1a associated with ERα transcript *in vivo*. (E-H) MCF-7 and (I-L) BT474 cell lysates were immunoprecipitated with RNPC1a antibody or control IgG followed by RT-PCR (E, I) and qRT-PCR (F-H, J-L) measure transcript levels of ERα, p21, HuR and Actin within RNPC1a or IgG immunocomplexes. Data were means of three separate experiments and performed as mean ± SEM, *p < 0.05, **p < 0.01.

Overexpression of RNPC1a in MCF-7 cells increased the level of ERα transcript. The the half-life of ERα transcript was increasd from 3.4 h to >8.0 h (Figure 3A), suggesting that ERα stability was regulated by RNPC1a. In BT474 cells, the half-life of ERα transcript was increased from 3.7 h to >8.0 h (Figure 3B). Moreover, the half-life of ERα transcript was decreased after RNPC1a knockdown. In MCF-7 cells, the half-life of ERα transcript was decreased from 5.8 h in control cells to 2.9 h in RNPC1a knockdown cells (Figure 3C). Similarly, the half-life of ERα transcript was decreased from 4.4 h in control BT474 cells to 2.7 h in RNPC1a knockdown BT474 cells (Figure 3D). Together, these data demonstrated that RNPC1a increased the stability of ERα transcript.

Then, we investigated whether RNPC1a physically associated with ERα transcript. RNA immunoprecipitation assay followed by RT-PCR (Figure 3E) and qRT-PCR (Figure 3F-H) was performed on extracts from MCF-7 cells. It showed that ERα transcript was present in RNPC1a, but not in the control IgG immunocomplexes (Figure 3E). P21 and HuR transcripts were positive controls as they had previously been deciphered to form immunocomplexes with RNPC1a. As a control, RNPC1a was unable to bind to Actin mRNA. Similarly in BT474 cells, ERα, p21 and HuR transcripts were also present in RNPC1a, but not in control IgG (Figure 3I-L). It indicated that RNPC1a could physically bind to ERα transcript.

### Multiple regions in the ERα 3′UTR were bound by RNPC1 and responsive to RNPC1

**Figure 4:**
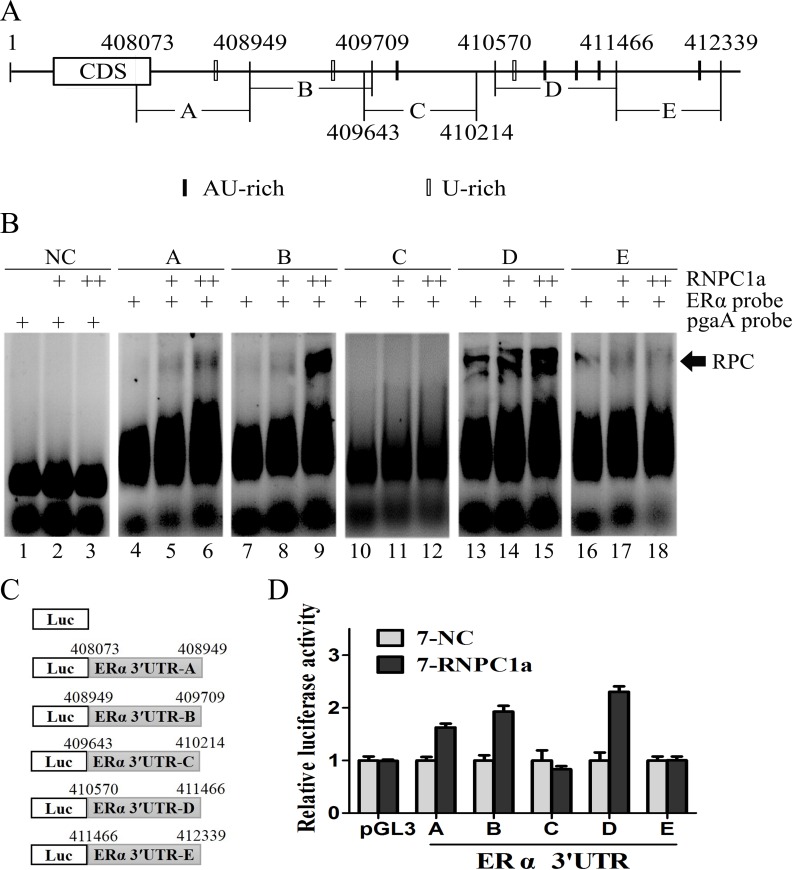
Multiple regions in the ERα 3′UTR were bound by RNPC1 and responsive to RNPC1 (**A**) Schematic representation of ERα transcript and the location of probes used for REMSA. The AU- or U-rich elements were shown in shaded boxes. (**B**) RNPC1a bound to multiple regions in ERα 3′-UTR. Probes A, B and D, but not probes C and E, associated with RNPC1a. REMSA was performed by mixing probe A, B, C, D or E with His-tagged RNPC1a protein, respectively. The density of His-tagged RNPC1a protein was 0.6μg (+) and 1.2μg (++). The negative control group (NC) was performed by mixing RNPC1a protein with probe pgaA, which couldn't combine with His-tagged RNPC1a protein. The bracket indicated RNA-protein complexes (RPC). (**C**) Schematic representation of the luciferase plasmid with various region of ERα 3′-UTR. (**D**) The luciferase activity for the reporter carrying ERα 3′UTR-A, -B or -D was increased by RNPC1a. MCF-7 cells with RNPC1a overexpression lentivirus (RNPC1a) and the control (NC) were transfected with pGL3 reporter carrying various regions of ERα 3′UTR for 48 h, respectively. Cells were then harvested for luciferase assay as described in ‘Materials and methods'. The fold increase in relative luciferase activity is a product of the luciferase activity induced by RNPC1a (7-RNPC1a) divided by that induced by an empty NC (7-NC) vector.

**Figure 5:**
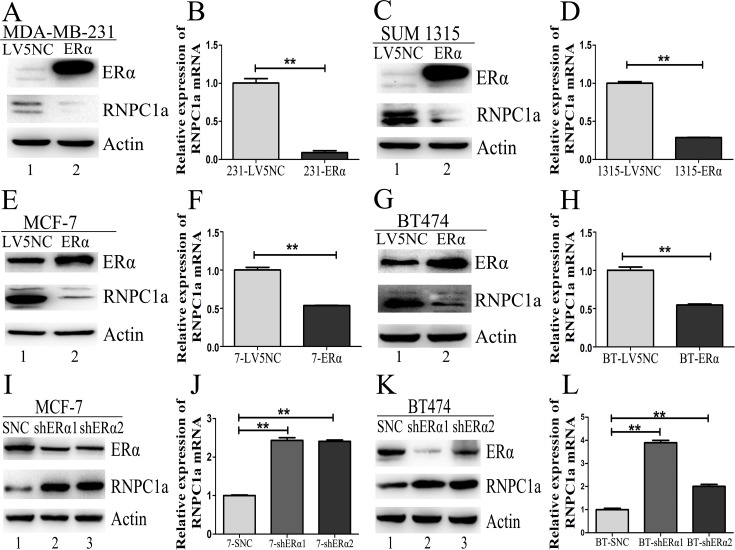
ERα could reversely regulate endogenous RNPC1 expression (A-D) The expression of RNPC1a was reduced by ERα overexpression in ER negative breast cancer cells. (A, B) MDA-MB-231 was transfected with ERα overexpression (ERα) and the control (LV5NC) lentivirus. (**A**) Western blot and (**B**) qRT-PCR were used to analyze the expression of ERα and RNPC1a. (C, D) The experiment shown in panel A was also performed in SUM 1315 cells. (**C**) Western blot and (**D**) qRT-PCR were used to analyze the expression of ERα and RNPC1a. (E-H) The expression of RNPC1a was reduced by ERα overexpression in ER positive breast cancer cells. (E, F) MCF-7 was transfected with ERα overexpression (ERα) and the control (LV5NC) lentivirus. (**E**) Western blot and (**F**) qRT-PCR were used to analyze the expression of ERα and RNPC1a. The experiment shown in panel E was also performed in BT474 cells. (**G**) Western blot and (**H**) qRT-PCR were used to analyze the expression of ERα and RNPC1a. (I-L) The expression of RNPC1a was increased with ERα knockdown in ER positive breast cancer cells. (I, J) MCF-7 was transfected with ERα knockdown (shERα1, shERα2) and the control (SNC) lentivirus. (**I**) Western blot and (**J**) qRT-PCR were used to analyze the expression of ERα and RNPC1a. (K, L) The experiment shown in panel I was also performed in BT474 cells. (**K**) Western blot and (**L**) qRT-PCR were used to analyze the expression of ERα and RNPC1a. The relative quantification was calculated by the ΔΔCt method and normalized based on β-actin. Data were means of three separate experiments and performed as mean ± SEM, **p < 0.01.

RNA electrophoretic mobility shift assay (REMSA) was performed to detect the binding site(s) of RNPC1a in ERα transcript. The recombinant His-tagged RNPC1a protein formed a complex with probe A, B and D, respectively (Figure 4B, comparing lanes 4, 7, 13 with 5, 8, 14, respectively), compared with the negative control (NC) (Figure 4B). Probes C and E were unable to connect with recombinant His-tagged RNPC1a (Figure 4B, comparing lane 10, 16 with 11, 17, respectively). The combination of RNA-protein was increased with protein density (Figure 4B, comparing lanes 5, 8, 14 with 6, 9, 15, respectively). It suggested that RNPC1a could bind to ERα mRNA 3′UTR. To functionally confirm the AU/U-rich elements were required for RNPC1a binding to the ERα transcript, we performed a dual-luciferase assay using pGL3 reporters that carried various region of ERα 3′UTR, including 3′UTR-A, B, C, D and E, whose sequences were identical to probes A, B, C, D and E, respectively (Figure 4C). The luciferase activity for a reporter carrying ERα 3′UTR-A, B and D was significantly increased by RNPC1a. By contrast, the ERα 3′UTR-C and E were not responsive to RNPC1a (Figure 5D). Taken together, these data suggested that ERα 3′UTR-A, B and D were responsive to RNPC1a and that each region was sufficient for RNPC1a to increase ERα expression.

### ERα reversely regulated endogenous RNPC1 expression

ER negative cells MDA-MB-231 and SUM 1315 were transfected with ERα overexpression lentivirus. The expression of RNPC1a and ERα in these stably infected cells was analyzed by Western blot (Figure 5A and 5C) and qRT-PCR (Figure 5B and 5D, both p < 0.01; Figure S3A and B, p < 0.01). The expression of RNPC1a was significantly deceased after ERα overexpression. Similar results were obtained in two ER positive cell lines MCF-7 and BT474 (Figure 5E, 5G, 5F and 5H, p < 0.01; Figure S3C and S3D, p < 0.01). It suggested that ERα could reversely down-regulate RNPC1a expression.

Conversely, we examined whether ERα knockdown could regulate RNPC1a expression. ER positive cells MCF-7 and BT474 were transfected with ERα knockdown lentivirus. The expression of RNPC1a and ERα in these stably infected cells was analyzed by Western blot (Figure 5I and 5K) and qRT-PCR (Figure 5J and 5L, both p < 0.01; Figure S3E and S3F, p < 0.01). The expression of RNPC1a was increased after ERα knockdown. Altogether, these data indicated ERα reversely regulated endogenous RNPC1a expression.

### Estrogen reduced the expression of endogenous RNPC1

**Figure 6:**
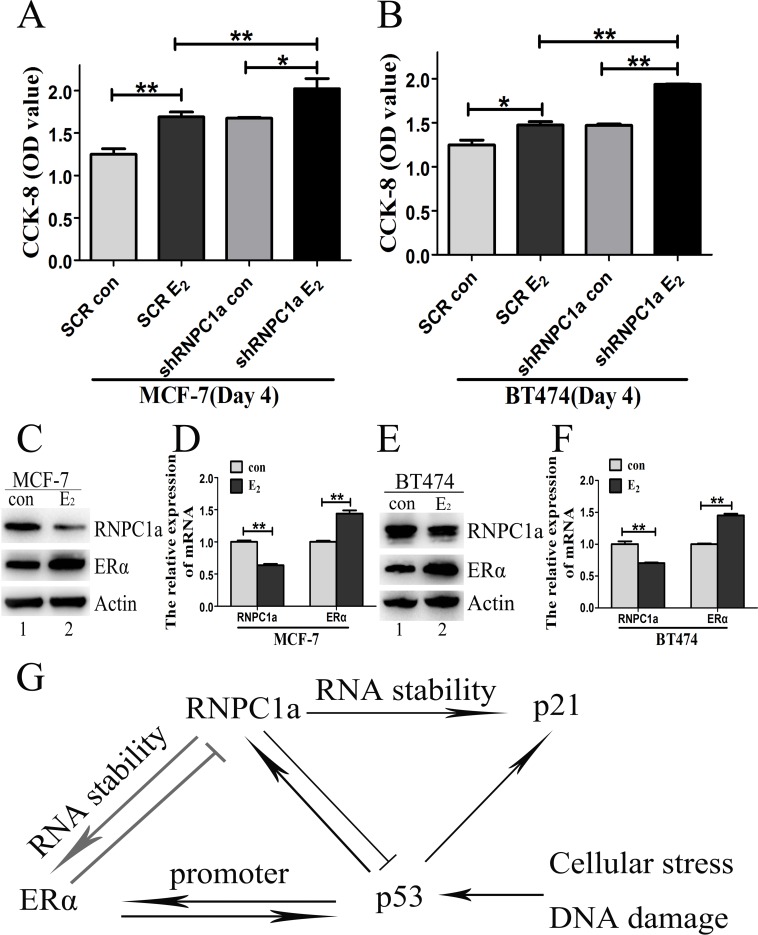
Estrogen reduced the expression of endogenous RNPC1 (A, B) The growth of RNPC1a knockdown cells was increased with estrogen (E_2_) treatment for 4 days. (**A**) The RNPC1a knockdown (shRNPC1a) and the control cells (SCR) were treated with estrogen treatment or not for 4 days in MCF-7. The growth of cells was measured by using cell counting kit (CCK-8) assays. The proliferation of RNPC1a knockdown cells was significantly increased compared with the control cells. (**B**) The experiment shown in panel A was also performed in BT474 cells. Data were means of three separate experiments and performed as mean ± SEM, *p < 0.05, **p < 0.01. (C, D) Estrogen reduced the expression of endogenous RNPC1a. (**C**) Western blot and (**D**) qRT-PCR were used to analyze the expression of ERα and RNPC1a followed by treated with estrogen for 48 h in MCF-7 cells. (E, F) The experiment shown in panel C was also performed in BT474 cells. (**E**) Western blot and (**F**) qRT-PCR were used to analyze the expression of ERα and RNPC1a. The relative quantification was calculated by the ΔΔCt method and normalized based on β-actin. Data were means of three separate experiments and performed as mean ± SEM, **p < 0.01. (**G**) A model for the interplay among RNPC1, ERα and p53. RNPC1a, a p53 family target, increased ERα expression by stabilizing its mRNA. Similarly, RNPC1a could promote p21 express via increasing its mRNA stability. ERα and p53 have a bi-directed relationship affecting both expression and function. RNPC1a, ERα and p53 exhibited complex interplay among them.

To explore the influence of estrogen on RNPC1a, the growth of RNPC1a knockdown cells and related control cells treated with estrogen or not for 4 days was determined by CCK-8 assay (Figure 6A and 6B). The proliferation rate of RNPC1a knockdown cells was obviously increased compared with the controls in MCF-7 (Figure 6A, p < 0.01) and BT474 (Figure 6B, p < 0.01) cells. To further examine the effect of estrogen on RNPC1a expression, MCF-7 and BT474 cells were treated with estrogen for 48 h. The expression of RNPC1a and ERα was analyzed by Western blot (Figure 6C and 6E) and qRT-PCR (Figure 5D and 5F, both p < 0.01). The expression of RNPC1a was decreased, while ERα increased. It indicated that estrogen could reduce the expression of endogenous RNPC1a.

## DISCUSSION

In the previous study, we found RNPC1 expression was silenced in breast cancer cell lines compared to breast epithelial cells. Moreover, RNPC1 was frequently silenced in breast cancer tissue compared to adjacent normal breast tissue. Functional assays showed ectopic expression of RNPC1 could inhibit breast tumor cell proliferation *in vivo* and *in vitro* through inducing cell cycle arrest, and suppress tumor cell migration and invasion [31]. Furthermore, RNPC1 showed strong relationship with ERα expression in breast cancer patients.

To our knowledge, ERα plays a critical role in breast cancer, such as classifying, prognosis, diagnosis, most importantly, target of endocrine therapy. In this study, we further confirmed RNPC1 was significantly correlated with ERα expression in breast cancer tissues. Overexpression of RNPC1 increased ERα expression in ER positive breast cancer cells. Conversely, RNPC1 knockdown decreased ERα expression in ER positive breast cancer cells. There was no change of ERα state in ER negative breast cancer cells, neither RNPC1 overexpression nor knockdown. Moreover, RNPC1 had no effect on the expression of ERβ. RNPC1 was found to be able to increase ERα stability by prolonging its half-life after treated with actinomycin D for various times, while RNPC1 knockdown obviously decreased the stability of ERα transcript. We further confirmed that RNPC1 could bind to ERα transcript directly by RIP (Figure 3E-L).

Previous studies found that RNPC1 could bind to mRNA 3′UTR of many genes and changed the stability of the transcripts [32-37]. The combined transcripts contain multiple AU/CU/U-rich elements [32-37]. Consistent with this, ERα 3′UTR contains several AREs, which can be bound by RBPs. In these studies, the main regions in the 3′UTR recognized by RNPC1 are principally multiple AU/U-rich elements (AREs). Our results indicated that RNPC1 was able to bind to multiple sites in the ERα 3′UTR, which were located in three regions, 3′UTR-A, B and D (Figure 4B). Binding of ERα ARE within its 3′-UTR by RNPC1 leads to enhanced stability of ERα transcript. Moreover, to confirm the function of sequence A, B and D, we showed that the luciferase activity for a reporter carrying each region can be obviously increased by RNPC1a. Our data indicate that RNPC1 is a positive post-transcriptional regulator of ERα. It is a novel mechanism by which ERα expression is regulated via mRNA stability, besides the post-translational mechanisms including phosphorylation, acetylation, sumoylation [28] and ubiquitin-proteasome pathway [29, 30].

When ERα overexpressed in breast cancer cells, RNPC1 expression was reversely reduced. While, the expression of RNPC1 increased after ERα knockdown. It indicated that there was a novel regulatory feedback loop between RNPC1 and ERα. Moreover, ER binds to specific DNA sequences called estrogen response elements (EREs) with high affinity and activates gene expression in response to estrogen. The classical ERα binding sites (EREs) is 5′-GGTCAnnnTGACC-3′, where n is any nucleotide [12-13]. However, we found there were not any EREs in the promoter of RNPC1 (data not shown). We implied ERα was indirectly involved in the regulation process of RNPC1 express. Long-term high estrogen stimulation could increase the incidence of breast cancer. We found the proliferation of RNPC1 knockdown cells increased after treatment of estrogen, compared with that of the control cells. While the expression of endogenous RNPC1 decreased with estrogen treatment. Altogether, these data indicated RNPC1 played an anti-tumor effect, which was consisted with our previous study [31].

Plenty data has shown that RNPC1, a target of p53 family, is a critical regulator of p53 translation [32, 37, 38]. Although tumor suppressor p53 is the most commonly mutated gene in most human cancers, it is only mutated in about 20% breast cancers. In our previous study, low RNPC1 expression was significantly associated with mutp53 in breast cancer tissues and acted as a tumor suppressor [31]. P53 can up-regulate ERα gene expression by binding to its ERα promoter [39, 40]. Meanwhile, p53, as a target gene of ERα, can be activated by ERα via binding to p53 promoter in ER positive breast cancers [41]. Therefore, ERα and p53 have a bi-directed relationship affecting both expression and function [39-41]. RNPC1 can reversely inhibit p53 expression via directly binding to p53 5′ and 3′ UTRs [37]. At the same time, p53 can up-regulate ERα gene expression by binding to its ERα promoter [39, 40]. It implies that RNPC1 may reduce ERα expression through an indirect way. The ectopically RNPC1a overexpression increased endogenous ERα expression by binding directly to 3′-UTR of ERα transcript and increasing its stability. More importantly, further study demonstrated ectopically ERα overexpression could reversely decrease endogenous RNPC1a expression. This demonstrated there was a feedback regulatory loop between RNPC1a and ERα expression. Based on these findings, we propose a model for the regulation of ERα by RNPC1 and the interplay among RNPC1, ERα and p53 (Figure 6G). Meanwhile, RNPC1 can enhance the stability of p21 and induce cell cycle arrest [38, 42]. Nevertheless, we found the overexpression of ERα in breast cancer could reversely repress RNPC1 expression, while the expression of RNPC1 increased followed by ERα knockdown. It is possible the ectopic of ERα can inhibit the regulation of RNPC1 on factors like p21 and then influence cell cycle.

In summary, as a target of endocrine therapy, the expression status of ER has an enormous significance on the prognosis of breast cancer. We revealed a novel mechanism by which ERα expression was regulated by RNA stability. A feedback loop between RNPC1 and ERα was described. RNPC1 expression may be an intriguing prognostic factor in ER positive breast cancer. These findings might provide highlights for ER regulation and targets for clinical endocrine-therapy strategy.

## MATERIALS AND METHODS

### Cell lines and cell culture

The human breast cancer cell lines MCF-7, BT474, MDA-MB-231 and SUM 1315 were obtained from American Type Culture Collection (ATCC, VA, USA). The cells were cultured in a humidified atmosphere of 5% CO_2_ at 37°C and fed with complete high glucose Dulbecco's modified Eagle medium (DMEM), supplemented with 10% fetal bovine serum, 1% penicillin–streptomycin solution. For 17β-estradiol treatment, MCF-7 and BT474 were cultured in DMEM without phenol red, supplemented with 5% steroid-depleted foetal bovine serum (BI, Israel) for 4 days prior to 17β-estradiol (estrogen, E_2_, Sigma, USA) treatment.

### Lentivirus transfection

Lentivirus constructs were generated to overexpress ERα. The breast cancer cells were stably transfected with ERα overexpression (termed as ERα) lentivirus and LV5-EF1a-GFP-Puro negative control vectors (termed as LV5NC), following the manufacturer's instructions (GenePharma, Shanghai, China). For ERα knockdown, the breast cancer cells were stably transfected with LV3-pGLV-h1-GFP-puro negative control vectors (termed as SNC) and ERα knockdown lentivirus (termed as shER1, shER2, shER3, shER4). Lentiviral constructs of RNPC1a overexpression and knockdown were generated as previously described [31]. Briefly, the breast cancer cells were stably transfected with RNPC1a overexpression lentivirus (termed as RNPC1a) and a negative control (termed as NC). The breast cancer cells were stably transfected with a negative control (termed as SCR) and RNPC1a knockdown lentivirus (termed as sh1, sh2, sh3). Cells were plated in 6 wells dishes at 30% confluence and infected with the retroviruses. Meanwhile, polybrene (5 μg/ml) was added with the retroviruses to enhance infection efficiency. Stable pooled populations of breast cancer cells were generated by selection using puromycin (3 μg/ml) for 2 weeks. For RNPC1a knockdown, one construct (sh2), named as shRNPC1a, with ≥85% knockdown efficiency was used for further studies. For ERα knockdown, two constructs (shER1, shER2), named as shERα1 and shERα2, with ≥85% knockdown efficiency was used for further studies.

### Western blotting analysis

The cells were seeded in 10×10 mm^2^ dishes. After treatment, cells were washed twice with cold phosphate buffer solution (PBS, Hyclone, USA) and then scraped off in 1000 μl lysis buffer containing 1% phenylmethanesulfonyl fluoride (PMSF) and 0.1 % protease inhibitor cocktail (KeyGen, Nanjing, China) and centrifuged at 14,000 g at 4°C for 15 min. The supernatants were obtained as total proteins and stored at −80°C for further studies. The total proteins were electrophoresed by 10-12% SDS-PAGE gel, and transferred to polyvinylidene fluoride (PVDF, Millipore, USA) membranes, which was activated in methanol. The blots were probed or reprobed with antibodies. The membranes were probed using Immobilon Western Chemiluminescent HRP Substrate (Millipore, USA) and autoradiographed. The intensity of the bands was determined using densitometric analysis. The primary antibodies used were anti-rabbit RBM38, the alia name of RNPC1, (Santa Cruz, USA), ERα (Cell Signaling technology, USA), ERβ (Cell Applications, USA), anti-mouse Actin (Cell Signaling technology, USA). The anti-rabbit and anti-mouse secondary antibodies were from Cell Signaling technology. Actin was used to normalize protein loading. The antibodies were diluted according to the manufacturer's instructions.

### RNA extraction, reverse transcription and quantitative RT-PCR (qRT-PCR)

Total RNA was isolated from cells using Trizol reagent (TaKaRa, Japan), and cDNA was synthesized using Primescript RT Reagent (TaKaRa, Japan) following manufacturer's instructions. The PCR program used for amplification was (i) 94°C for 30 seconds, (ii) 94°C for 30 seconds, (iii) 55°C for 30 seconds, (iv) 72°C for 1 minute, and (v) 72°C for 10minutes. From steps 2 to 4, the cycle was repeated 35 times for β-actin and other genes. To amplify all the genes, the following PCR primers were used:

RNPC1a forward, 5′-ACGCCTCGCTCAGGAA GTA-3′

RNPC1a reverse, 5′-GTCTTTGCAAGCCCTCT CAG-3′

β-actin forward, 5′-GCTGTGCTATCCCTGTAC GC-3′

β-actin reverse, 5′-TGCCTCAGGGCAGCGGAA CC-3′

ERα forward, 5′-CCCACTCAACAGCGTGTC TC-3′

ERα reverse, 5′-CGTCGATTATCTGAATTTGGC CT-3′

ERβ forward, 5′-AGCACGGCTCCATATACATAC C-3′

ERβ reverse, 5′-TGGACCACTAAAGGAGAAAGG T-3′

P21 forward, 5′-TGTCCGTCAGAACCCATGC-3′

P21 reverse, 5′-AAAGTCGAAGTTCCATCGC TC-3′

HuR forward, 5′-AACTACGTGACCGCGAAGG-3′

HuR reverse, 5′-CGCCCAAACCGAGAGAACA-3′

All PCR reactions were performed using the fluorescent SYBR Green I methodology. Quantitative RT-PCR (qRT-PCR) was performed on StepOnePlus Real-Time PCR system (Applied Biosystems, USA) using FastStart Universal SYBR Green Master (Roche, Switzerland) according to the manufacturer's instructions. The qRT-PCR conditions consisted of an initial denaturation step at 95°C for 10 minutes, followed by 40 cycles of 15 seconds at 95°C and 1 minute at 60°C. A melting curve was set at 95°C for 15 seconds, 60°C for 15 seconds, and 95°C for 15 seconds at the end of each run to verify the specificity. The relative quantification was calculated by the ΔΔCt method and normalized based on β-actin.

### Immunofluorescence (IF)

The immunofluorescence was used to verify the expression location of RNPC1a and ERα. Briefly, the breast cancer cells were plated in 24-well plate at a density of 5×10^4^ cells/well and incubated for 12h. After washed with phosphate-buffered saline (PBS, pH=7.4) twice, the cells were fixed with paraformaldehyde for 20 min and penetrated by 0.5% Tritonx-100 for 10 min, followed by blocking for 1 h in blocking buffer. Then cells were incubated with primary antibody overnight at 4 °C. After washed with PBS three times, cells were incubated for 1 h in the dark with FITC-conjugated secondary goat anti-rabbit antibodies (Invitrogen, USA). The cells were then washed and stained with 4, 6-diamidino-2-phenylindole (DAPI) for 5 min. Immunostaining was observed under a Zeiss fluorescence microscope at 400× magnification.

### Cell counting kit (CCK-8) assay

Cell proliferation was assessed by using CCK-8 kit (Dojindo, Japan) according to the manufacturer's instruction. Briefly, 2×10^3^ cells were seeded into a 96-well plate in triplicate and 8 hours later 17β-estradiol (Estrogen, E_2_) was added into the wells at the concentration of 1×10^−7^ M, while cells cultured in medium with 0.01% DMSO. On the days of measuring the growth rate of cells, the medium in each well was replaced with 100 μl fresh medium containing 10% CCK-8. The pates were incubated at 37 °C for 3 h and then read at 450 nm with a microplate reader (5082 Groding, Tecan, Austria). All tests were performed in triplicate.

### Tissue samples

The breast cancer sample tissue microarrays (BC08118) for immunohistology analysis (IHC) were purchased from Biomax (USA). Histologic types were classified according to the World Health Organization (2003). TNM staging was defined according to the American Joint Committee on Cancer (AJCC) (the 6th version, 2002). All the cases were individually categorized by two independent pathologists.

### Immunohistochemical (IHC) staining

The IHC staining of the tissue microarrays was performed as previously described [43-44]. The same tissue samples were stained with RNPC1a and ERα antibody respectively. The RBM38 antibody (LifeSpan Biosciences, USA) was used at the dilution of 1:350. The ERα antibody (Cell Signaling technology, USA) was used at the dilution of 1:300. The rabbit polyclonal antibody was used as anti-RBM38 and ERα primary antibody.

### Analysis of immunochemistry

The breast cancer tissues were scored semiquantitatively on the basis of a well-established immunoreactivity scoring system (IRS) [45]. The final staining results of RNPC1a and ERα were described as follows. Firstly, the staining intensity (SI) was scored on a scale of 0-3. The score 0 was attained for totally negative cases. For weak, moderate, and strong staining, the scores were 1, 2 and 3, respectively. Secondly, the percentage of positive cells (PP) was scored into five categories: no staining, 1-10, 11-50, 51-80, 81-100 percentage positive cells. And the scores were 0, 1, 2, 3 and 4, respectively. An immunoreactivity scoring system (IRS) was calculated by multiplying the percentage of positive cells (PP) times the staining intensity (SI) score, resulting in a scale from 0 to 12. The IRS was divided into three groups: negative (IRS 0-3), or low staining (IRS 4-7) and high staining (IRS 8-12). The tissue microarrays were observed under 200× magnification.

### RNA immunoprecipitation (RIP)

The breast cancer cells (2×10^7^) were lysed with RNA immunoprecipitation lysis buffer (Millipore, USA) and then incubated with 5 μg of rabbit polyclonal anti-RBM38 (Santa Cruz Biotechnology, USA) or non-immunized rabbit IgG at 4°C overnight. The RNA-protein immunocomplexes were brought down by protein A/G magnetic beads, followed by RNA purification. After that, the purified RNA was subjected to RT-PCR and qRT-PCR. The primers to detect human ERα, p21 and HuR mRNA expressions were the same as those described previously.

### Recombinant protein purification and RNA probes

*E. coli* BL21 (DE3) was transformed with a pET28a vector expressing His-tagged RNPC1a and positive clones were selected. After induction by isopropyl β-D-1-thiogalactopyranoside (IPTG), the recombinant proteins were then purified by Ni-NTA beads (Sepharose^TM^ 6 Fast Flow, GE Healthcare, UK), following the manufacturer's instructions. To generate RNA electrophoretic mobility shift assay (REMSA) probes, various regions (A-E) in ERα 3′-UTR were amplified by PCR. The T7 promoter sequence was introduced into one terminus of the PCR products with primers, which were listed as follows.

The primers for probe A were:5′-GGATCCTAATACGACTCACTATAGGGAGGAGCTCCCTGGCTCCCACACGGTTC-3′ and 5′- ACTGGAACAGGTCCTGAAGCTGACCTTAC-3′.

The primers for probe B were:5′-GGATCCTAATACGACTCACTATAGGGAGTGGGCACTGTACTTGGATCTTC-3′ and 5′- TCACCCAGAGGAAATCAAACATTC-3′.

The primers for probe C were:5′-GATCCTAATACGACTCACTATAGGGAGCTCTAGCACAATTATGGGTTAC-3′ and 5′- CACCAGGCTTTAGGCATAAATGAC-3′.

The primers for probe D were:5′-GGATCCTAATACGACTCACTATAGGGAGATGTGTTTCTATTCATGTTAAGATAC-3′ and 5′- ACAGTCCATCTCATAATTGGAAAGTATG-3′.

The primers for probe E were:5′-GGATCCTAATACGACTCACTATAGGGAGTGGGTACTGGGAGTGATCACTAACAC-3′ and 5′- AATTGTTTACAGGTGCTCGAGCATC-3′.

The primers for the pgaA were: 5′-CTGAGCTCAGGCATTGGGATTTATGCCGT-3′ and 5′-GACTCGAGCACCTTTTTCTGCTACTTGAATAC-3′.

### Probe labeling and RNA electrophoretic mobility shift assay (REMSA)

REMSA was performed as Zhang et al [33] with some modification [46]. Briefly, the PCR products of various regions from A to E were used as the templates for RNA preparation, using the T7 RNA polymerase (Thermo, USA). Templates were then digested with RNase-free DNase I (TaKaRa, Japan) and RNA probes were purified with RNeasy Mini Kit (Qiagen, Germany). Probes were incubated with His-tagged RNPC1a in a 20 μl volume containing 10 mmol/L HEPES-KOH (pH 7.5), 90 mmol/L potassium acetate, 1.5 mmol/L magnesium, 2.5 mmol/L dithiothreitol (DTT) and 40 U of RNase inhibitor at 30°C for 30 min. The reaction employing RNPC1 protein and the *pgaA* probe was used as a negative control [47]. To prevent non-specific binding, 2.5μg yeast tRNA (Ambion, USA) was added in the reaction system. RNA-protein complexes were resolved on a 4 % agarose gel and detected by UV transillumination after Gel Red staining.

### Luciferase assay

Dual-luciferase reporter assay was performed in triplicate according to manufacturer's instructions (Promega, USA). Briefly, 5ng of Renilla luciferase vector (pRL-CMV; Promega, USA), an internal control, and 200ng of a pGL3 reporter which contained various region of ERα 3′UTR were co-transfected into MCF-7 RNPC1a overexpression (7-RNPC1a) and the control (7-NC) cells. Forty-eight hours after transfection, luciferase activity was measured with the dual luciferase kit according to manufacturer's procedure (Promega, USA). The fold change in relative luciferase activity is a ratio of the luciferase activity induced by 7-RNPC1a divided by that induced by 7-NC.

### Statistical analysis

The data were analyzed using the SPSS 20.0 software. All experiments in this study were repeated in triplicate, unless otherwise specified. The χ^2^ test was used to assess the correlation between RNPC1a and the clinicopathological parameters. The linear correlation analysis was used to assess the correlation between RNPC1a and ERα. For all the continuous variables, Student t-test and two-way ANOVA were used to analyze the statistical significance of the differences between groups, and P<0.05 was considered to indicate a statistically significant difference.

## SUPPLEMENTARY MATERIAL AND FIGURES



## References

[1] DeSantis CE, Lin CC, Mariotto AB, Siegel RL, Stein KD, Kramer JL, Alteri R, Robbins AS, Jemal A (2014). Cancer treatment and survivorship statistics, 2014. CA: a cancer journal for clinicians.

[2] Siegel R, Ma J, Zou Z, Jemal A (2014). Cancer statistics, 2014. CA: a cancer journal for clinicians.

[3] Santen RJ, Boyd NF, Chlebowski RT, Cummings S, Cuzick J, Dowsett M, Easton D, Forbes JF, Key T, Hankinson SE, Howell A, Ingle J, Breast Cancer Prevention Collaborative G (2007). Critical assessment of new risk factors for breast cancer: considerations for development of an improved risk prediction model. Endocrine-related cancer.

[4] Key T, Appleby P, Barnes I, Reeves G, Endogenous H, Breast Cancer Collaborative G (2002). Endogenous sex hormones and breast cancer in postmenopausal women: reanalysis of nine prospective studies. Journal of the National Cancer Institute.

[5] Caldon CE (2014). Estrogen signaling and the DNA damage response in hormone dependent breast cancers. Frontiers in oncology.

[6] Preston-Martin S, Pike MC, Ross RK, Jones PA, Henderson BE (1990). Increased cell division as a cause of human cancer. Cancer research.

[7] Ascenzi P, Bocedi A, Marino M (2006). Structure-function relationship of estrogen receptor alpha and beta: impact on human health. Molecular aspects of medicine.

[8] Ali S, Coombes RC (2000). Estrogen receptor alpha in human breast cancer: occurrence and significance. Journal of mammary gland biology and neoplasia.

[9] Schneider AE, Karpati E, Schuszter K, Toth EA, Kiss E, Kulcsar M, Laszlo G, Matko J (2014). A dynamic network of estrogen receptors in murine lymphocytes: fine-tuning the immune response. Journal of leukocyte biology.

[10] Treeck O, Lattrich C, Springwald A, Ortmann O (2010). Estrogen receptor beta exerts growth-inhibitory effects on human mammary epithelial cells. Breast cancer research and treatment.

[11] Speirs V, Carder PJ, Lane S, Dodwell D, Lansdown MR, Hanby AM (2004). Oestrogen receptor beta: what it means for patients with breast cancer. The Lancet Oncology.

[12] Moggs JG, Orphanides G (2001). Estrogen receptors: orchestrators of pleiotropic cellular responses. EMBO reports.

[13] Jordan VC (2004). Selective estrogen receptor modulation: concept and consequences in cancer. Cancer cell.

[14] Pearce ST, Jordan VC (2004). The biological role of estrogen receptors alpha and beta in cancer. Critical reviews in oncology/hematology.

[15] Renoir JM, Marsaud V, Lazennec G (2013). Estrogen receptor signaling as a target for novel breast cancer therapeutics. Biochemical pharmacology.

[16] Khan SA, Rogers MA, Khurana KK, Meguid MM, Numann PJ (1998). Estrogen receptor expression in benign breast epithelium and breast cancer risk. Journal of the National Cancer Institute.

[17] Liedtke C, Broglio K, Moulder S, Hsu L, Kau SW, Symmans WF, Albarracin C, Meric-Bernstam F, Woodward W, Theriault RL, Kiesel L, Hortobagyi GN, Pusztai L, Gonzalez-Angulo AM (2009). Prognostic impact of discordance between triple-receptor measurements in primary and recurrent breast cancer. Annals of oncology : official journal of the European Society for Medical Oncology / ESMO.

[18] Pusztai L, Viale G, Kelly CM, Hudis CA (2010). Estrogen and HER-2 receptor discordance between primary breast cancer and metastasis. The oncologist.

[19] Yao ZX, Lu LJ, Wang RJ, Jin LB, Liu SC, Li HY, Ren GS, Wu KN, Wang DL, Kong LQ (2014). Discordance and clinical significance of ER, PR, and HER2 status between primary breast cancer and synchronous axillary lymph node metastasis. Medical oncology.

[20] Normanno N, Di Maio M, De Maio E, De Luca A, de Matteis A, Giordano A, Perrone F, Group NC-NBC (2005). Mechanisms of endocrine resistance and novel therapeutic strategies in breast cancer. Endocrine-related cancer.

[21] Hussain SA, Palmer DH, Moon S, Rea DW (2004). Endocrine therapy and other targeted therapies for metastatic breast cancer. Expert review of anticancer therapy.

[22] Heldring N, Pike A, Andersson S, Matthews J, Cheng G, Hartman J, Tujague M, Strom A, Treuter E, Warner M, Gustafsson JA (2007). Estrogen receptors: how do they signal and what are their targets. Physiological reviews.

[23] Meneses-Morales I, Tecalco-Cruz AC, Barrios-Garcia T, Gomez-Romero V, Trujillo-Gonzalez I, Reyes-Carmona S, Garcia-Zepeda E, Mendez-Enriquez E, Cervantes-Roldan R, Perez-Sanchez V, Recillas-Targa F, Mohar-Betancourt A, Leon-Del-Rio A (2014). SIP1/NHERF2 enhances estrogen receptor alpha transactivation in breast cancer cells. Nucleic acids research.

[24] Merot Y, Metivier R, Penot G, Manu D, Saligaut C, Gannon F, Pakdel F, Kah O, Flouriot G (2004). The relative contribution exerted by AF-1 and AF-2 transactivation functions in estrogen receptor alpha transcriptional activity depends upon the differentiation stage of the cell. The Journal of biological chemistry.

[25] Levin ER, Pietras RJ (2008). Estrogen receptors outside the nucleus in breast cancer. Breast cancer research and treatment.

[26] Qin C, Samudio I, Ngwenya S, Safe S (2004). Estrogen-dependent regulation of ornithine decarboxylase in breast cancer cells through activation of nongenomic cAMP-dependent pathways. Molecular carcinogenesis.

[27] Razandi M, Pedram A, Rosen EM, Levin ER (2004). BRCA1 inhibits membrane estrogen and growth factor receptor signaling to cell proliferation in breast cancer. Molecular and cellular biology.

[28] Anbalagan M, Huderson B, Murphy L, Rowan BG (2012). Post-translational modifications of nuclear receptors and human disease. Nuclear receptor signaling.

[29] Wang Y, Zong H, Chi Y, Hong Y, Yang Y, Zou W, Yun X, Gu J (2009). Repression of estrogen receptor alpha by CDK11p58 through promoting its ubiquitin-proteasome degradation. Journal of biochemistry.

[30] Cheng L, Li J, Han Y, Lin J, Niu C, Zhou Z, Yuan B, Huang K, Li J, Jiang K, Zhang H, Ding L, Xu X, Ye Q (2012). PES1 promotes breast cancer by differentially regulating ERalpha and ERbeta. The Journal of clinical investigation.

[31] Xue JQ, Xia TS, Liang XQ, Zhou W, Cheng L, Shi L, Wang Y, Ding Q (2014). RNA-binding protein RNPC1: acting as a tumor suppressor in breast cancer. BMC cancer.

[32] Yan W, Zhang J, Zhang Y, Jung YS, Chen X (2012). p73 expression is regulated by RNPC1, a target of the p53 family, via mRNA stability. Molecular and cellular biology.

[33] Zhang J, Jun Cho S, Chen X (2010). RNPC1, an RNA-binding protein and a target of the p53 family, regulates p63 expression through mRNA stability. Proceedings of the National Academy of Sciences of the United States of America.

[34] Yin T, Cho SJ, Chen X (2013). RNPC1, an RNA-binding protein and a p53 target, regulates macrophage inhibitory cytokine-1 (MIC-1) expression through mRNA stability. The Journal of biological chemistry.

[35] Cho SJ, Jung YS, Zhang J, Chen X (2012). The RNA-binding protein RNPC1 stabilizes the mRNA encoding the RNA-binding protein HuR and cooperates with HuR to suppress cell proliferation. The Journal of biological chemistry.

[36] Xu E, Zhang J, Chen X (2013). MDM2 expression is repressed by the RNA-binding protein RNPC1 via mRNA stability. Oncogene.

[37] Zhang J, Cho SJ, Shu L, Yan W, Guerrero T, Kent M, Skorupski K, Chen H, Chen X (2011). Translational repression of p53 by RNPC1, a p53 target overexpressed in lymphomas. Genes & development.

[38] Shu L, Yan W, Chen X (2006). RNPC1, an RNA-binding protein and a target of the p53 family, is required for maintaining the stability of the basal and stress-induced p21 transcript. Genes & development.

[39] Shirley SH, Rundhaug JE, Tian J, Cullinan-Ammann N, Lambertz I, Conti CJ, Fuchs-Young R (2009). Transcriptional regulation of estrogen receptor-alpha by p53 in human breast cancer cells. Cancer research.

[40] Angeloni SV, Martin MB, Garcia-Morales P, Castro-Galache MD, Ferragut JA, Saceda M (2004). Regulation of estrogen receptor-alpha expression by the tumor suppressor gene p53 in MCF-7 cells. The Journal of endocrinology.

[41] Berger CE, Qian Y, Liu G, Chen H, Chen X (2012). p53, a target of estrogen receptor (ER) alpha, modulates DNA damage-induced growth suppression in ER-positive breast cancer cells. The Journal of biological chemistry.

[42] Cho SJ, Zhang J, Chen X (2010). RNPC1 modulates the RNA-binding activity of, and cooperates with, HuR to regulate p21 mRNA stability. Nucleic acids research.

[43] Schindlbeck C, Jeschke U, Schulze S, Karsten U, Janni W, Rack B, Krajewski S, Sommer H, Friese K (2007). Prognostic impact of Thomsen-Friedenreich tumor antigen and disseminated tumor cells in the bone marrow of breast cancer patients. Breast cancer research and treatment.

[44] Beck T, Weikel W, Brumm C, Wilkens C, Pollow K, Knapstein PG (1994). Immunohistochemical detection of hormone receptors in breast carcinomas (ER-ICA, PgR-ICA): prognostic usefulness and comparison with the biochemical radioactive-ligand-binding assay (DCC). Gynecologic oncology.

[45] Honing J, Pavlov KV, Meijer C, Smit JK, Boersma-van Ek W, Karrenbeld A, Burgerhof JG, Kruyt FA, Plukker JT (2014). Loss of CD44 and SOX2 expression is correlated with a poor prognosis in esophageal adenocarcinoma patients. Annals of surgical oncology.

[46] Lin PC, Xu RM (2012). Structure and assembly of the SF3a splicing factor complex of U2 snRNP. The EMBO journal.

[47] Wang X, Dubey AK, Suzuki K, Baker CS, Babitzke P, Romeo T (2005). CsrA post-transcriptionally represses pgaABCD, responsible for synthesis of a biofilm polysaccharide adhesin of Escherichia coli. Molecular microbiology.

