# Supplementary material for: Estrogen receptor (ER) was regulated by RNPC1 stabilizing mRNA in ER positive breast cancer
Source: Oncotarget. 2015 Mar 26;6(14):12264–78. doi: 10.18632/oncotarget.3654 (PMC4494937; doi:10.18632/oncotarget.3654)
Supplement: Supplementary file 1 [file oncotarget-06-12264-s001.pdf]

Estrogen receptor (ER) was regulated by RNPC1 stabilizing mRNA in ER positive breast cancer

Supplementary Material

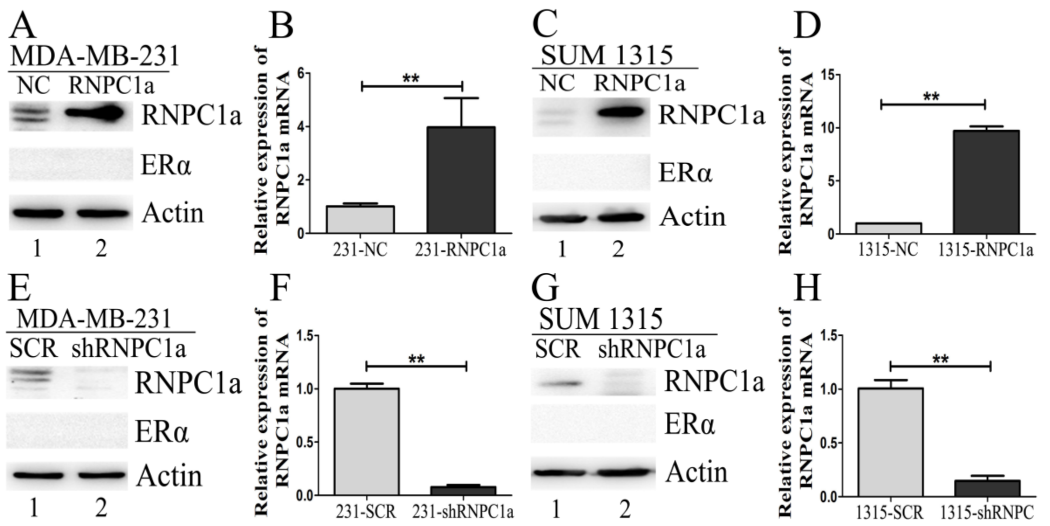

Figure S1: RNPC1 couldn't affect ERα expression in ER negative breast cancer cells.

(A, B, C, D) The expression of ERα was not influenced by RNPC1a overexpression. (A, B) MDA-MB-231 was transfected with lentivirus containing either control luciferase (NC) or RNPC1a overexpression (RNPC1a). (A) Western blot and (B) qRT-PCR were used to analyze the expression of RNPC1a and ERα. (C, D) The experiment shown in panel A was also performed in SUM 1315 cells. (C) Western blot and (D) qRT-PCR were used to analyze the expression of RNPC1a and ERα. (E, F, G, H) The expression of ERα was not influenced by RNPC1a knockdown. (E, F) MDA-MB-231 was transfected with a control (SCR) and RNPC1a knockdown (shRNPC1a) lentivirus. (E) Western blot and (F) qRT-PCR were used to analyze the

expression of RNPC1a and ERα. (G, H) The experiment shown in panel E was also performed in SUM 1315 cells. (G) Western blot and (H) qRT-PCR were used to analyze the expression of RNPC1a and ERα. The relative quantification was calculated by the △△Ct method and normalized based on β-actin. Data were means of three separate experiments and performed as mean ± SEM, **$p < 0.01$.

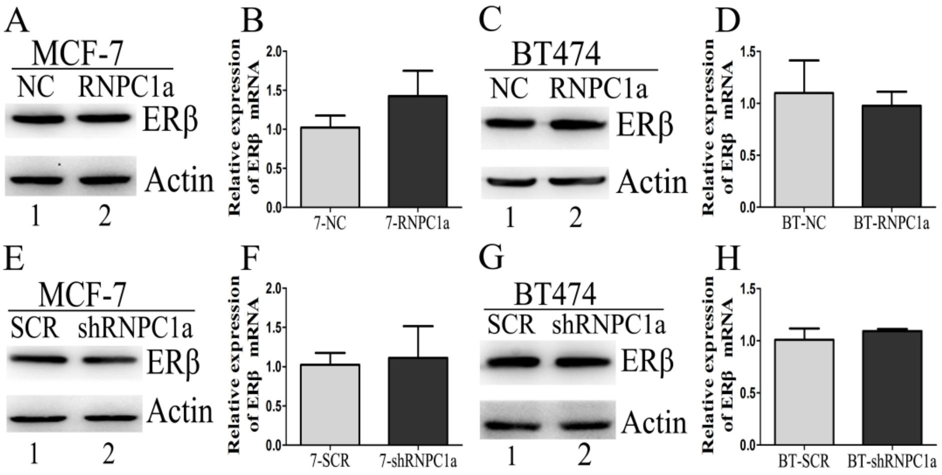

Figure S2: ERβ expression was not affected by RNPC1a.

(A, B, C, D) The expression of ERβ was not influenced by RNPC1a overexpression. (A, B) MCF-7 was transfected with a control (NC) and RNPC1a overexpression (RNPC1a) lentivirus. (A) Western blot and (B) qRT-PCR were used to analyze the expression of ERβ. (C, D) The experiment shown in panel A was also performed in BT474 cells. (C) Western blot and (D) qRT-PCR were used to analyze the expression of ERβ. (E, F, G, H) The expression of ERβ was not affected by RNPC1a knockdown. (E, F) MCF-7 was transfected with a control (SCR) and RNPC1a knockdown (shRNPC1a) lentivirus. (E) Western blot and (F) qRT-PCR were used to analyze the expression of ERβ. (G, H) The experiment shown in panel E was also performed in BT474 cells. (G) Western blot and (H) qRT-PCR were used to analyze the expression of ERβ. The relative quantification was calculated by the △△Ct method and normalized based on β-actin. Data were means of three separate experiments and performed as mean ± SEM.

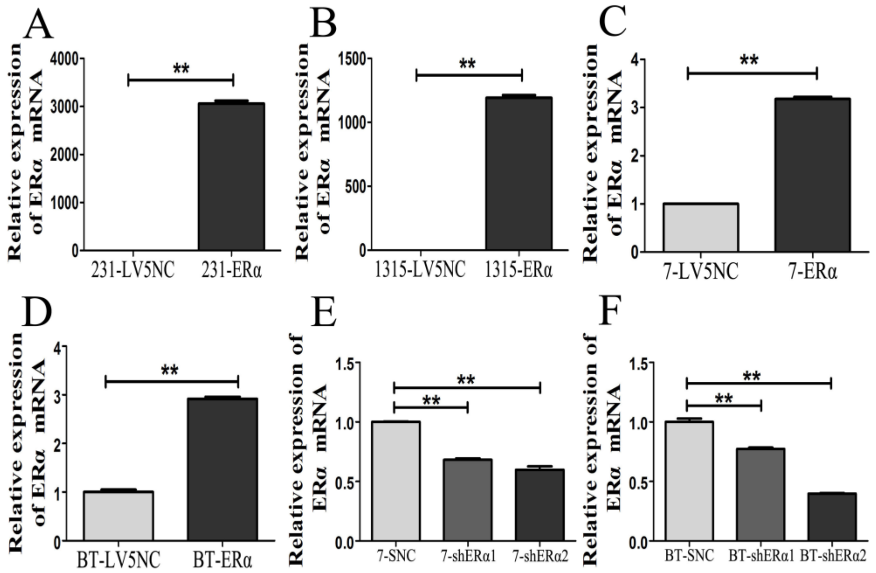

Figure S3: ERα lentivirus was successfully transfected into breast cancer cells.

(A, B) ERα was expressed in ER negative breast cancer cells after transfection. (A) MDA-MB-231 and (B) SUM 1315 were transfected with ERα overexpression (ERα) and the control (LV5NC) lentivirus. qRT-PCR were used to analyze the expression of ERα in the cells. (C, D) ERα was expressed in ER positive breast cancer cells after transfection. (C) MCF-7 and (D) BT474 were transfected with ERα overexpression (ERα) and the control (LV5NC) lentivirus. qRT-PCR were used to analyze the expression of ERα in the cells. (E, F) ERα was knocked down in ER positive breast cancer cells after transfection. (E) MCF-7 and (F) BT474 were transfected with ERα knockdown (shERα1, shERα2) and the control (SNC) lentivirus. qRT-PCR were used to analyze the expression of ERα in the cells. The relative quantification was calculated by the $\Delta\Delta$Ct method and normalized based on β-actin. Data were means of three separate experiments and performed as mean ± SEM, **$p < 0.01$.
